# Evaluation of Pacific White Shrimp (*Litopenaeus vannamei*) Health during a Superintensive Aquaculture Growout Using NMR-Based Metabolomics

**DOI:** 10.1371/journal.pone.0059521

**Published:** 2013-03-26

**Authors:** Tracey B. Schock, Jessica Duke, Abby Goodson, Daryl Weldon, Jeff Brunson, John W. Leffler, Daniel W. Bearden

**Affiliations:** 1 Chemical Sciences Division, National Institute of Standards and Technology, Hollings Marine Laboratory, Charleston, South Carolina, United States of America; 2 Marine Resources Research Institute, South Carolina Department of Natural Resources, Charleston, South Carolina, United States of America; Oak Ridge National Laboratory, United States of America

## Abstract

Success of the shrimp aquaculture industry requires technological advances that increase production and environmental sustainability. Indoor, superintensive, aquaculture systems are being developed that permit year-round production of farmed shrimp at high densities. These systems are intended to overcome problems of disease susceptibility and of water quality issues from waste products, by operating as essentially closed systems that promote beneficial microbial communities (biofloc). The resulting biofloc can assimilate and detoxify wastes, may provide nutrition for the farmed organisms resulting in improved growth, and may aid in reducing disease initiated from external sources. Nuclear magnetic resonance (NMR)-based metabolomic techniques were used to assess shrimp health during a full growout cycle from the nursery phase through harvest in a minimal-exchange, superintensive, biofloc system. Aberrant shrimp metabolomes were detected from a spike in total ammonia nitrogen in the nursery, from a reduced feeding period that was a consequence of surface scum build-up in the raceway, and from the stocking transition from the nursery to the growout raceway. The biochemical changes in the shrimp that were induced by the stressors were essential for survival and included nitrogen detoxification and energy conservation mechanisms. Inosine and trehalose may be general biomarkers of stress in *Litopenaeus vannamei*. This study demonstrates one aspect of the practicality of using NMR-based metabolomics to enhance the aquaculture industry by providing physiological insight into common environmental stresses that may limit growth or better explain reduced survival and production.

## Introduction

In 2011, shrimp was the leading seafood import in the United States, accounting for approximately 28% of all seafood imports, and total expenditures on shrimp imports exceeded $5 billion [1]. Approximately 91% of the U.S. shrimp supply is imported and aquaculture is responsible for 55% of global shrimp production [2], [3]. Compared to the world’s leading producers, primarily Southeast Asian and South American industries, the U.S. shrimp aquaculture industry is disadvantaged by the United States’ temperate climate (which is unfavorable for year-round shrimp production), higher costs for land, labor, and other inputs, and stricter environmental and food quality regulations [4], [5].

Improving and expanding the U.S. shrimp aquaculture industry offers many economic, social, and environmental benefits. Production of more shrimp in the U.S. could reduce expenditures on shrimp imports, increase revenue from exports, and potentially create new jobs. Domestically grown shrimp would also give U.S. producers and consumers more control over the quality of shrimp, and improving shrimp aquaculture practices in general could diminish the industry’s global ecological footprint.

Superintensive, minimal-exchange culturing systems represent a promising option for the U.S. aquaculture industry. Minimal to zero-exchange systems reduce pollution discharge, water usage, and risk of disease exchange between wild and captive stocks. Superintensive stocking reduces habitat destruction by limiting land use and increases economic competitiveness through higher yields per crop cycle[6]–[8]. Furthermore, the use of greenhouse-enclosed raceway ponds allow U.S. aquaculturists to overcome climate disadvantages by maintaining necessarily warm water temperatures as well as reduce disease initiated from external sources [9], [10].

These types of aquaculture systems are dependent on a dense microbial community to maintain water quality by coupling removal of excess feed and toxic chemicals from the water with production of biomass that can be used as a supplemental nutritional source for shrimp. This microbial community resides primarily on suspended “biofloc” particles comprised of shrimp feed, fecal matter, and detritus [8]–[11].

When considering the tolerance of Pacific white shrimp, *Litopenaeus vannamei*, to low salinities, these aquaculture schemes are particularly enticing because they could be positioned inland and provide fresh marine shrimp to areas that lack such a resource naturally while reducing time to market and transportation costs [7]. Overall, use of these types of systems would allow for increased production throughout the U.S.

Maintaining ideal growth conditions in superintensive, minimal-exchange, biofloc-based systems requires careful management that often relies on, for example, oxygen injection and regulation of the concentration of suspended biofloc particles through the use of settling chambers[6]–[8]. The biofloc itself is a dynamic growth medium in which imbalances in microbial species or nutrient concentrations can impair growth rates or cause shrimp mortality [6], [8]. The density of the microbial biofloc also makes it impossible to visibly observe the shrimp to assess their behavior and health.

As a result, a growing demand exists for a technique to quickly and efficiently detect stressors in a culture environment before shrimp health and growth are severely affected. Metabolomics is a systems biology approach to the study of metabolites and application of this research field has grown rapidly in recent years, particularly in the realm of human health. Metabolomics holistically assesses organismal health through the examination of metabolic profiles of biofluids and tissues that reflect the physiological state of the organism. Perturbations to the “normal/healthy” state due to stressors (diet, toxicity, infection, disease, or environmental factors) can be detected in the subtle changes of the metabolic profile resulting in potential biomarkers of the stress. NMR spectroscopy-based metabolomics represents an especially promising technique for aquaculture because it offers a reproducible, quantitative, non-destructive method for high-throughput sample analysis, allowing for characterization of a large number of samples at relatively low incremental cost per sample and short data collection times[12]–[15]. Relatively few metabolomic aquaculture studies have been published and all pertain to fish species[12], [16]–[23]. Here we present a production analysis of an important U.S. commodity (shrimp) using this versatile technique that has the ability to provide a wealth of information to the aquaculture community.

NMR-based metabolomics was used to analyze Pacific white shrimp (*Litopenaeus vannamei*) health during a 72 day nursery and 126 day growout cycle conducted at the South Carolina Department of Natural Resources’ Waddell Mariculture Center (WMC), Bluffton, SC, during which growth, nutritional components, water quality, biofloc composition, and other characteristics of the system were monitored. The objective of the study was to assess NMR-based metabolomics as a diagnostic tool to shrimp aquaculture for detecting stressors in the culture environment and for evaluating shrimp health. The capability to detect the onset of stress or disease in an aquaculture system can lead to improved system management by identifying and correcting suboptimal environmental conditions that might occur during nursery and growout phases. Nutritional deficiencies that might arise at different stages of shrimp growth can also be identified and addressed through improved formulations and dietary regimes [12]. Such advances are essential to the improvement and expansion of the industry.

## Methods

**Figure 1:**
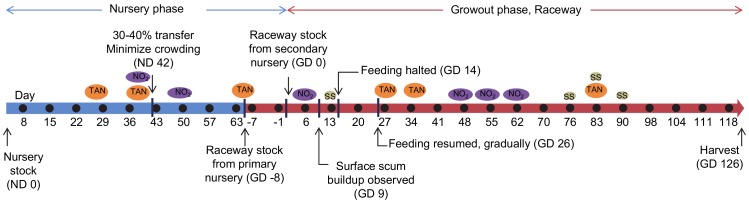
Growout timeline. Experimental design of the shrimp aquaculture growth study from the nursery phase through the raceway growout phase to harvest. Circles represent shrimp collection days, •: biological composites (nursery) or shrimp muscle tissue (growout) were analyzed. ND: nursery phase day; GD: growout phase day. Important events are noted with arrows and text. Water quality changes are illustrated as colored circles above the timeline, TAN: a spike in total ammonia nitrogen levels; NO_2_: a spike in nitrite levels, SS: an increase in suspended solids.

A detailed schematic of the experimental design is illustrated in Figure 1. Important events that occurred during the growout are highlighted in this figure and specific details are described throughout the manuscript.

### Shrimp Husbandry

#### Nursery phase

On August 10, 2010, *L. vannamei* postlarvae (PL 12) were obtained from Shrimp Improvement Systems (Islamorada, FL) and stocked into a 42.9 m^2^ (41.8 m^3^) ethylene propylene diene Monomer (M-class) rubber (EPDM; Firestone Specialty Products, Indianapolis, IN) lined nursery tank, constructed of concrete blocks, and housed in a greenhouse at the WMC. The tank had been filled with Colleton River (Bluffton, SC) water passed through a sand filter, 10 µ and 5 µ bag filters, and an ultraviolet sterilizer. Postlarvae (PL) were stocked at a density of 5981 PL/m^3^, and were fed newly hatched *Artemia* for 3 days. *Shrimp PL Raceway Plus* crumble diet (Ziegler Bros., Inc., Gardners, PA) was fed according to a predetermined feeding protocol regularly used at WMC, with larger size, lower protein and fat feeds gradually introduced as the shrimp grew. Shrimp were fed by hand four times per day during the nursery phase of production. The nursery was operated as an enclosed minimal exchange, biofloc-based system, with water exchanges occurring only as needed to maintain water quality and replace evaporative loss. Primary aeration was supplied by a 3728.49 W (5 hp) regenerative blower, which also provided aeration for other culture systems. Pure oxygen was required to maintain optimal levels of dissolved oxygen as the oxygen demand in the system increased, and was supplied from a liquid oxygen source through ultra-fine pore diffusers.

Temperature, dissolved oxygen, salinity and pH were measured twice daily using a YSI 556 Multiparameter meter (YSI Incorporated, Yellow Springs, OH). All other parameters were measured weekly: total ammonia nitrogen (TAN; Hach Method 8155, Hach DR/4000V spectrometer, Hach Company, Loveland, CO), nitrite nitrogen (NO_2_-N; Hach Method 8507), nitrate-nitrogen (NO_3_-N; Hach Method 8039), orthophosphate (Dionex ICS-2000 ion chromatograph, Thermo Fisher Scientific, Inc., Sunnyvale, CA), alkalinity (Mettler Toledo DL28 titrator, Mettler-Toledo, LLC, Columbus, OH) and turbidity (LaMotte 2020e turbidimeter, LaMotte Company, Chestertown, MD). Occasional ammonia and nitrite spikes were controlled with water exchange, or with the addition of dextrose, a carbon source to stimulate heterotrophic bacteria that assimilate inorganic nitrogen.. During these periods, water quality parameters were tested at more frequent intervals using the stated methods. Regular additions of sodium bicarbonate were used to maintain alkalinity between 100 mg L^−1^ CaCO_3_ and 150 mg L^−1^ CaCO_3_. Individual weight of the postlarval and juvenile shrimp (n = 100) was sampled weekly. Excess water was removed by allowing the PLs and juveniles to dry on paper towels for a short time (<5 minutes) before weighing.

On September 21, 2010, day 42 of the nursery phase (ND 42), 30% to 40% of the shrimp were transferred from the primary nursery tank in order to minimize stress and water quality deterioration due to crowding. The secondary nursery tank to which they were moved had a bottom surface area of 30.5 m^2^, and a volume of 30 m^3^. The feeding regime in the secondary nursery tank was the same as that of the primary tank, and water quality was monitored in the same way.

The prototype commercial-scale growout raceway (271 m^2^, 235 m^3^) has a center divider and the water is circulated slowly by 56 airlifts that mix the water as it flows. In addition to the airlifts, oxygen can be generated and injected directly into the water to support higher densities of shrimp. A propane boiler and heat exchanger heat the water for year-round production. Sixteen days in advance of stocking with juveniles, the raceway had been filled with Colleton River (Bluffton, SC) water that passed through a sand filter, 10 µm and 5 µm bag filters, and an ultraviolet sterilizer. Small amounts of crushed shrimp feed were added to the raceway to stimulate bacterial activity and algal development. Eight days prior to stocking the juveniles from the primary nursery tank into the production raceway, water was slowly recirculated between the nursery tank and the raceway to facilitate a gradual acclimation of the shrimp to a new environment. Shrimp from the primary nursery were stocked on October 13, 2010, day (−8) of the growout phase (GD −8), using established techniques based on mean weight and the total weight stocked [24].

Although shrimp in the secondary nursery tank could not be acclimated by recirculating water from the raceway, efforts were made to match the temperature, pH and salinity to that of the production raceway immediately prior to stocking of the juveniles. Differences in water quality parameters between the secondary nursery and the raceway were minor (1.2°C temperature difference, 1.45 ppt salinity difference, 0.02 pH unit difference). Shrimp stocking from the secondary nursery occurred on October 21, 2010, using the same stocking techniques. This date was considered Day 0 of the growout cycle in the production raceway (GD 0). An estimated 89,066 shrimp from the primary nursery and 32,634 from the secondary nursery were stocked. Assuming a 1.5% mortality of the first group between October 13 and 21, the relative weights of both the primary and secondary groups were measured on October 21, and a composite number and biomass calculated. On Day 0 of the growout cycle the raceway was stocked at 512 m^−3^ with a mean individual weight of 2.90 g.

#### Growout phase

The commercial-scale shrimp production raceway at the WMC is designed to be a minimal exchange, superintensive system, wherein microfauna and flora, either unattached, or attached to suspended particles (biofloc), tank walls and structures within the system, process waste products from feed metabolism by shrimp. Of particular interest from a shrimp health perspective are ammonia and nitrite which are toxic to shrimp [25]. A biofloc-based system depends upon its microbial community to metabolize these waste products rapidly enough to maintain concentrations that support shrimp growth and health. While suspended solids are essential to provide substrate for the microbial community, an excess amount of solids creates a high oxygen demand and may reduce shrimp growth rates [6], [7]. Solids “cropping” was achieved by pumping water into a 1150 L conical bottom settling chamber, where water flowed down through a 25.4 cm corrugated pipe suspended in the center of the chamber and then slowly rose back up to an overflow pipe that returned the water to the raceway (Figure 1 in Ray *et al.* [7]). Since the water flowed back out of the chamber approximately 15 cm below the surface, both settleable and floating solids were removed. Collected solids were drained from the settling chamber once or twice a week, as needed. The settling chamber was operated in an attempt to hold total suspended solids between 300 mg L^−1^ and 500 mg L^−1^.

As described above, temperature, dissolved oxygen, salinity and pH were measured twice daily and TAN, NO_2_-N, NO_3_-N, orthophosphate, alkalinity, and turbidity were measured weekly. Total and volatile suspended solids (TSS, VSS; APHA Standard Methods 2540-D, 2540-E) and chlorophyll *a* (USEPA Method 446.0) were also measured weekly during the growout phase[26]–[28]. During times of elevated TAN or NO_2_-N levels, measurement occurred more frequently so that corrective measures could be taken. If necessary, dextrose was added to control ammonia and nitrite concentrations by stimulating heterotrophic bacteria. Alkalinity was maintained between 100 mg L^−1^ CaCO_3_ and 150 mg L^−1^ CaCO_3_ by regular additions of sodium bicarbonate.

Shrimp in the raceway were hand-fed a commercially produced, pelleted ration (Zeigler Bros. Inc. *Shrimp Grower Hyper-Intensive 35 EX*) 3 times daily. Feed amount was calculated based on a targeted individual growth rate of 1.0 gram per week and a feed conversion ratio (FCR) of 1.6 (i.e., 1.6 g of feed to produce 1 g of shrimp tissue), and adjusted weekly for an expected mortality rate of 1.5% per week. Feeding rate was further adjusted based on observations of shrimp behavior and consumption from feeding trays, as well as water quality parameters and other environmental conditions. Ten collections of 25 shrimp each were sampled and weighed weekly to monitor growth rate. Harvest of the raceway occurred on February 24, 2011, on day 126 of the growout cycle (GD 126).

### Shrimp Collection for Metabolomics

#### Nursery collection

During the nursery phase, shrimp were collected weekly from August 18, 2010 (ND 8) to October 6, 2010 (ND 57) at approximately the same time of day. Each week, five replicate nursery samples were collected. Each sample was a biological composite consisting of one to five shrimp, limited by the number that could be placed in a 5 mL cryovial. For these collections, shrimp were netted, rinsed with sterile seawater, blotted on a paper towel, placed in a labeled cryovial with forceps, and immediately frozen in liquid nitrogen. The collected samples were stored at −80°C.

#### Raceway collection

Individual shrimp from the nursery were sampled one day prior to transfer to the raceway, October 12, 2010 (ND 63), one day after the transfer, October, 14, 2010 (GD −7), and then weekly until the final sampling before harvest, February 16, 2011 (GD 118). At each collection, 10 whole shrimp were sampled, 10 shrimp were dissected for the hepatopancreas and tail muscle, and 5 shrimp were harvested for intestines. Twenty shrimp (10 whole and 10 for dissection) were individually netted, rinsed with sterile seawater, and immersed in liquid nitrogen for approximately 15 s. The whole shrimp were placed in a cryovial, a 15 mL tube, or a plastic bag depending on size and were kept at −20°C for several weeks then transferred to −80°C for long term storage. The frozen shrimp for dissection were sectioned between the 1^st^ and 2^nd^ abdominal segments. The carapace was removed from the thorax and the hepatopancreas was excised. Tail muscle was taken from the third abdominal segment. Intestines were collected from five freshly caught shrimp. The intestine was collected by bisecting the shrimp by gently pinching between the thorax and abdomen, and delicately pulling the intestine from the abdomen. Each tissue sample was stored in a cryovial in liquid nitrogen for several weeks and transferred to −80°C for long-term storage. The hepatopancreas and intestine samples were not analyzed.

#### Feed and biofloc collection

At the time of weekly collections, beginning from the nursery through the final harvest, samples of the feed being fed at the time were collected in a cryovial and stored at −80°C. Water samples (≈70 ml total per sample) containing suspended biofloc were also collected weekly in two 50 mL centrifuge tubes which were flash frozen in liquid nitrogen and stored at −80°C for analysis at a later time.

### Metabolomics Tissue Extraction

#### Nursery extraction

Each biological composite sample consisting of 1 to 5 PL or juvenile shrimp (n = 43) was lyophilized overnight (77% mass fraction water) and homogenized to a fine powder with a chemically cleaned mortar and pestle. Approximately 24 mg of dried composite shrimp homogenate (100 mg wet weight equivalent) was extracted using a chloroform:methanol:water technique modified from Wu and colleagues [29]. The dry tissue was pulverized using a Precellys 24 homogenizer (Bertin Technologies, France) at 523.59 rad s^−1^ (5000 rpm) for 15 s in a bead-beater tube with ceramic beads (2.8 mm). A polar solvent mix consisting of cold methanol (4 mL g wet mass^−1^ (gwm)) and water (1.6 mL gwm^−1^) was added to the homogenate in the bead beater tube. The tissue and polar solvents were homogenized at 680.68 rad s^−1^ (6500 rpm) for 15 s, two times. The whole polar homogenate was transferred to a glass vial containing cold chloroform (4 mL gwm^−1^) and water (2 mL gwm^−1^) for a final solvent volume ratio of 2 chloroform: 2 methanol: 1.8 water. The mixture was vortexed for 30 s, then incubated on ice for 10 min. The solvent phases were partitioned by centrifugation at 2000×*g*_n_ at 4°C for 5 min. The upper polar phase was removed with a pipette, and dried by Vacufuge (3 h, at room temperature; Eppendorf, Germany) and stored at −80°C until analyzed. The nonpolar portion was separated from the protein layer using a pulled-glass pipette pushed through the protein layer and stored in a glass vial at −80°C for analysis at a later time.

#### Raceway tissue extraction

Tail muscle tissue was extracted using a procedure similar to that for the nursery composites. Briefly, 100 mg of frozen tail tissue was placed in bead beating tubes and lyophilized overnight; shrimp muscle tissue is approximately 80% water by mass. The extraction process was performed as above.

#### Quality control material extraction

Technical variation was evaluated throughout the extraction process, particularly because the extractions took place over a few months with large time periods in between and different personnel involved. To ensure extraction technique reproducibility, a NIST standard reference material (SRM 1946– Lake Superior Fish Tissue) was extracted alongside all shrimp tissues (whole body (biological composites), and muscle tissue; n = 30 SRM samples processed). Additionally, control materials matrix matched to the test materials were produced by pooling 3^rd^ segment tail muscle tissues harvested from *L. vannamei*. The tail muscle was lyophilized then homogenized in a mortar and pestle. A total of 30 shrimp muscle control material (CM) samples were extracted during this study. Eight experimental samples were extracted in triplicate as technical replicates and six blanks (no tissue) were extracted as a contaminant control. The results of this analysis are reported in the Figure S1. To assess the analytical repeatability of our methods, the SRM extracts and the pooled muscle CM extract spectra were evaluated for spectral relative standard deviation (RSD) [30]. The analysis of SRM extracts resulted in a median %RSD of 12.4% with an interquartile range of 12.5%. The muscle CM spectra resulted in a median %RSD of 9.25% with an interquartile range of 12.8%. These median %RSD’s represent a high degree of control on the analytical precision of the extraction and measurement process. The eight triplicate analyses of random samples from the experimental sample set resulted in an average of the median %RSD of 7.7% (range from 5.4% to 10.4%).

### NMR Spectroscopy

The dried polar extracts were rehydrated with 600 µL 100 mmol L^−1^ phosphate buffer in D_2_O, pH 7.3, which included 1.0 mmol L^−1^ TMSP (3-Trimethylsilyl 2,2,3,3-d_4_ propionate, CAS 24493-21-8). Samples were reconstituted in sets of approximately 20 to minimize potential sample change/degradation from prolonged exposure to light and room temperature. The samples were analyzed using a Bruker Advance II 700 MHz spectrometer (Bruker Biospin, Inc., Billerica, MA) fitted with a cryogenically cooled probe (TCI 5 mm triple-resonance, z-gradient). Spectra were collected under automation with ICON-NMR with a one-dimensional (1D) ^1^H and a two-dimensional (2D) ^1^H J-resolved (JRES) pulse sequence. The 1D pulse sequence includes water suppression based on a three-pulse noesygppr1d which contains a spoiler gradient with 8 steady state scans, 40 transients, a 3 s relaxation delay, and a 60 ms mixing period. The data were acquired at 298 K with 65536 real data points across a spectral width of 20 ppm. The spectra were processed by multiplying the free induction decay by an exponential line broadening function of 0.3 Hz and the data were zero-filled to 65536 complex points prior to Fourier transformation (FT). The spectra were manually phased and the baseline was automatically corrected.

Two-dimensional edited heteronuclear single quantum correlation (HSQC) spectra with adiabatic ^13^C decoupling (HSQCEDETGPSISP2.2) were collected for selected samples to enhance metabolite identification. A relaxation delay equal to 1.5 s was used between acquisitions and a refocusing delay of 3.45 ms was implemented. In general, 2048 data points with 128 scans per increment were acquired with spectral widths of 11 ppm in F2 and 180 ppm in F1 (^13^C). The FIDs were weighted using a shifted sine bell function in both dimensions. Manual two-dimensional phasing was applied.

Shrimp metabolites were assigned based on 1D ^1^H and 2D ^1^H-^13^C NMR experiments. Assignments were based on comparison of chemical shifts and spin-spin couplings with reference spectra and tables such as those of the human metabolome database (HMDB) [31], the Madison metabolomics consortium database [32], the biological magnetic resonance data bank (BMRB) [33], and an in-house compiled database, as well as the SBASE-1-1-2 and bbiorefcode_0_1_2 databases used with AMIX (version 3.9.11; Bruker Biospin, Inc., Billerica, MA) and Chenomx® NMR Suite profiling software (version 7.12).

### Statistical Analysis

#### Multivariate data analysis

Principal components analysis (PCA) was performed to identify physiological changes in shrimp during an aquaculture growout. Processed ^1^H NMR spectra were analyzed with AMIX 3.9.ll statistical tools. The spectra from 0.5 ppm to 10.0 ppm, excluding the regions of the residual water resonance (4.7 ppm to 5.0 ppm), residual chloroform from the extraction process (7.67 ppm to 7.69 ppm) and a contaminant from one brand of bead beating tubes during muscle extractions (3.59 ppm to 3.61 ppm), were reduced to 1897 (nursery) and 1894 (muscle) bins 0.005 ppm wide. Signal intensities were summed for integration and the spectra were normalized to constant total spectral area. Prior to analysis, the data were mean-centered and pareto scaled. Individual PCA plots were constructed for nursery and raceway growout.

#### ^1^H significant difference spectra

Metabolites varying significantly in response to an aquaculture event or due to growth were identified by a univariate analysis of the binned spectra resulting in an informative one-dimensional biomarker profile termed ^1^H significant difference spectra (SDS) [12], [34]. Binned spectra were averaged by grouping (i.e., pre-event, event, or post-event) and, in a pairwise comparison, the averaged bin differences were calculated; for example the pre-event averaged bins were subtracted from the event averaged bins. Significantly changing bins were identified by a Student’s t-test. SDS were created by plotting the intensity differences of only the significantly different bins [35]. SDS spectra were visually evaluated for falsely identified bins of significance, such as adjacent bins with opposite signs and isolated single bins; the bins were removed from the generated SDS.

The levels of change in significant metabolites identified with SDS were assessed by calculating the percent change in response to the event or growth over time. Metabolite peaks of abundance and/or those well separated from other metabolite peaks were integrated using the AMIX multi-integrate tool. Peak integrals were normalized to TMSP (1.0 mmol L^−1^, at 0 ppm) and the resultant peak areas were exported to Excel to evaluate the percent change from the event (pre-event versus event) and to evaluate percent change of metabolites with time (pre-event versus post-event).

## Results and Discussion

Sustainable aquaculture requires a system that has good water quality, minimal impact on the environment, utilizes effective nutrition practices, and improves crops by enhancing immunity and limiting disease. Metabolomics analysis was applied to superintensive Pacific white shrimp, *Litopenaeus vannamei*, aquaculture with the goal of enhancing future production by identifying physiological stresses and/or beneficial conditions and biochemically explaining how these situations affect growth and survival.

### Superintensive Shrimp Aquaculture Production

During the 72-day nursery phase the mean water temperature in the primary nursery averaged 28.7°C ±1.4°C (mean ± SD) and 28.1°C ±1.7°C in the secondary nursery; dissolved oxygen averaged 6.4 mg L^−1^±1.4 mg L^−1^ and 5.0 mg L^−1^±0.9 mg L^−1^ respectively; and pH averaged 7.56±0.34 and 7.35±0.29 respectively. Salinity in the primary nursery started at 33.5 g L^−1^, the salinity at which the PLs arrived. A water exchange at ND 29 lowered the salinity to 30 mg L^−1^. Between ND 51 to 53, salinity was lowered to 19 g L^−1^ in preparation for stocking into the growout raceway at that concentration. Similarly, salinity started at 30.5 g L^−1^ in the secondary nursery and was reduced to 21.5 g L^−1^ prior to stocking. TAN concentration peaked to at least 12.9 mg L^−1^ around ND 29 before a complete water exchange over the next seven days brought it to 5.4 mg L^−1^ (ND 36) and 7.6 mg L^−1^ (ND 41) before dropping to 2.1 mg L^−1^(ND 42). NO_2_-N peaked at 10.35 mg L^−1^ on ND 49. These water quality parameters are summarized in Table S1.

Over the course of the raceway growout phase the mean water temperature averaged 28.6°C ±0.9°C (mean ± SD), dissolved oxygen averaged 10.2 mg L^−1^±1.8 mg L^−1^, pH averaged 7.19±0.37, and salinity averaged 18.9 g L^−1^±1.1 g L^−1^. Despite the addition of 308.9 kg of sodium bicarbonate, the pH of the raceway declined steadily from 8.4 to 6.7 over the course of the growout phase. Control of nitrogen wastes was not as efficient as expected. TAN concentrations peaked three times during the course of the growout at 5.3 mg L^−1^ (ND 63), 8.2 mg L^−1^ (GD 33), and 5.4 mg L^−1^ (GD 84). Nitrite-nitrogen peaked twice at 7.3 mg L^−1^ (GD 6) and 12.3 mg L^−1^ (GD 61).

After 126 days of growout, the raceway harvest produced 1,324 kg wet weight of heads-on shrimp, at a density of 5.63 kg m^−3^ with a mean shrimp weight of 18.83 g. Survival rate was 59% with a mean growth rate of 0.89 gram per week. The FCR for the growout phase was 2.0. In terms of expected production, these results are sub-optimal given that our goal for this production trial was to achieve 80% survival with a growth rate of 1.5 grams per week and a FCR of 1.6. The inferior performance might be attributed to stress resulting from periods of high ammonia and nitrite concentrations and a period of unusually high surface scum that was addressed by severely restricting feeding for several weeks.

### Metabolomics Analysis

#### NMR analysis

Due to the small size of the nursery shrimp (postlarval stage 13–70), whole-body biological composites were analyzed until the shrimp were stocked into the growout raceway, at which time they were approximately 2.0 g to 3.0 g and muscle tissue from individual shrimp was easily dissectible. Representative NMR spectra from a nursery-phase biological composite sample and a growout-phase shrimp muscle tissue show numerous spectral differences (Figure S2) due to metabolic compartmentalization in specific tissues. Spectra were annotated based on both 1D and 2D NMR experiments, resulting in over 50 specific metabolites. Many of the compounds identified are amino acids. Proline, arginine and glycine are reported to comprise greater than 70% (µmoles/g tissue wet weight) of the total free amino acid content in marine crustacean muscle tissue [36]. Other metabolite groups identified in these spectra (Table S2) include organic osmolytes (e.g., betaine and trimethylamine oxide (TMAO)), carbohydrates (e.g., glucose and trehalose), organic acids (e.g., lactate and acetoacetate), nucleosides (e.g., inosine and uridine) and energy phosphates (e.g., AMP, ADP and ATP).

#### Statistical analysis

Shrimp NMR spectra were assessed by principal component analysis (PCA), a method that facilitates graphical separation of groups according to overall metabolic fingerprints and is based on unsupervised analysis requiring no *a priori* classification of the data set. PCA reduces the high dimensionality of multivariate data while capturing most of the variance within a few dimensions [37]. The principal components are displayed graphically as a scores plot that is useful for observing any groupings in the data.

#### Nursery phase

**Figure 2:**
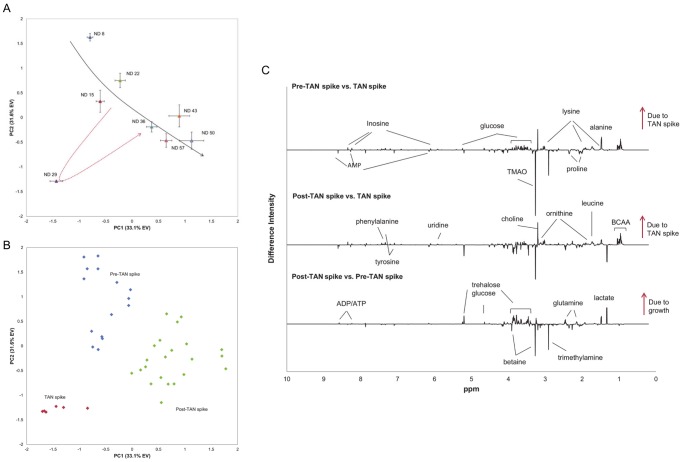
Nursery multivariate statistical analysis. Principal component analysis (PCA) scores plot of nursery phase shrimp. (A) Each point represents the mean PC score for the sampling time point (ND: nursery phase day) and the error bars represent the standard error of the mean. The solid black arrow suggests metabolic changes in relationship to growth, while the dashed red arrow denotes a deviation from and a return to “normal” growth. (B) PCA scores plot of all nursery phase shrimp grouped by pre-event, event (TAN spike), and post event. (C) ^1^H significant difference spectra (SDS) displaying metabolite changes in response to the TAN spike and growth. The top SDS spectrum compares pre-TAN spike vs. TAN spike, the middle spectrum compares TAN spike vs. post-TAN spike and the bottom spectrum compares pre-TAN spike vs. post-TAN spike. Metabolites increasing as a result of the TAN spike are positive in the top two spectra and metabolites increasing during growth are positive in the bottom spectrum. Not all compounds mentioned in the text are annotated here.

Forty-three whole body biological composite samples were analyzed by PCA for the 63 day growth during the nursery phase. The scores plot of PC1 vs. PC2 (65% of the variance explained) showed a general metabolic trend with growth, but a deviation from this trend occurred on ND 29 (Figure 2A). The aberrant metabolomes corresponded to a large rise in TAN which spiked from less than 1.0 mg L^−1^ to 12.9 mg L^−1^. This was remedied by a slow total tank volume (100%) water exchange over a seven-day period (ND 29 to ND 36) and the shrimp seemed to recover metabolically (Figure 2A). The TAN spike resulted in a growth rate of only 0.1 grams per week ±0.01 grams per week for the week following the spike. On a growth curve (Figure S3A) of the nursery samples this impact on production is barely visible and may typically be overlooked as harmful to production in practice, especially as the quality of the water improves and growth resumes. These results highlight the sensitivity of metabolomics measurements.

Metabolite changes in response to the TAN spike were identified by calculating ^1^H significant difference spectra (SDS) from the binned spectra [12] comparing metabolomes prior to the TAN spike (ND 8 to ND 22), the TAN spike event (ND 29), and all the shrimp harvested during the nursery phase after the water exchange (ND 36 to ND 57; Figure 2B and 2C). The patterns identified by calculating an SDS based on the pre-TAN spike event samples versus the TAN spike event samples (Fig 2C, upper, TAN_spike_ minus TAN_pre_) showed an increase in nearly all simple amino acids (lysine, arginine, asparagine, methionine, tyrosine and phenylalanine, ornithine, threonine, serine, alanine and tryptophan), an increase in branched-chain amino acids (BCAA, comprised of valine, isoleucine, and leucine), an increase in nucleosides (guanosine, uridine, and inosine, 2′- deoxyadenosine), and an increase in choline, maltose and glucose, but a decrease in AMP, ADP/ATP, NAD+, UDP-galactose, UDP-glucose, and trehalose in response to the TAN spike. As the corrective action (100% water exchange) returned the system to normal operating conditions, we could see changes in the SDS profile calculated from the TAN spike event samples versus the post-TAN spike event samples (Figure 2C, middle, TAN_spike_ minus TAN_post_) corresponding to the TAN spike-mediated metabolite changes similar to the analysis above. To understand the changes associated with a “normal growth” trajectory, an SDS contrasting the pre-TAN spike event samples and post-TAN spike event samples (Fig 2C, bottom, TAN_post_ minus TAN_pre_) showed alanine, lactate, glutamine, arginine, glucose, maltose and trehalose, and ADP/ATP all at increased concentrations, while glutamate, histidine, proline, pyroglutamate, sarcosine, dimethylamine, trimethylamine, 3-hydroxykynurenine, and acetoacetate all decreased with growth period.

**Figure 3:**
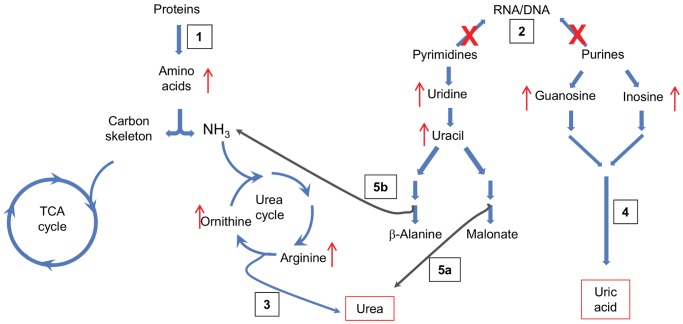
Affected biochemical pathways. Schematic depiction of the biochemical pathways *L. vannamei* may use to avoid NH_3_ toxicity during the TAN spike in the nursery phase. Blue arrows indicate normal metabolism pathways, red symbols indicate interrupted pathways or increasing concentrations due to the TAN spike, gray arrows denote pathways of microbial origin. (1) Increase in protein catabolism; (2) Inhibition of RNA and DNA synthesis; (3) Urea excretion through the OUC; (4) Uric acid excretion by purine catabolism; (5) Urea excretions by (a) pyrimidine oxidation and/or (b) pyrimidine reduction.

To evaluate the degree of change in metabolite levels with response to the TAN spike or during the nursery growout, the relative percent change was calculated for significant metabolites (Table S3 column (a) and (b), Figure S4). The large changes in amino acids, nucleosides and nucleobases (purines and pyrimidines) are evidence of biochemical mechanisms used to remove or avoid the accumulation of toxic ammonia (NH_3_). In intensive aquaculture systems, NH_3_ build up is the result of shrimp excretion and bacterial transformation of organic detritus like feces and unconsumed food and is highly toxic to production organisms. Shrimp bioaccumulate NH_3_ by diffusion which has caused retarded growth [38], [39], increased oxygen consumption [40], [41] and mortality [42]. In normal environmental conditions nitrogen is excreted mainly as NH_3_ (60% to 70% of total nitrogen) and amino acids (10%), with small amounts of urea and uric acid [43], [44]. In elevated NH_3_ environments, organisms decrease their NH_3_ excretion[41], [45]–[47] and may limit or completely stop feeding to reduce the internal accumulation of nitrogen waste products [39]. This may be a cause of the slowed growth rate seen in this stage of the study. The elevated TAN levels in the nursery resulted in high levels of amino acids, purines and pyrimidines in the metabolic profile suggesting increased macromolecular catabolism (breakdown of proteins; Figure 3(1)) and inhibition of nucleic acid synthesis (Figure 3(2)) in order to conserve energy and mobilize these compounds for storage as a nitrogen reserve. This process may also be a cause of the reduced growth. In addition, the catabolic process may be a mechanism to avoid NH_3_ toxicity by converting amino acids, purines and pyrimidines to urea or uric acid. Urea is formed through the hydrolysis of arginine in the ornithine-urea cycle (OUC; Figure 3(3)), and uric acid is produced by the degradation of purines (Figure 3(4)) [43], [44]. These alternative excretion pathways are active during the TAN spike event based on the observation of increased ornithine and the increased purines, inosine and guanosine (Table S3(a)), in the metabolic profile. These urea and uric acid excretion pathways have been documented for crustaceans in elevated NH_3_ environments with enzymatic and endpoint test studies [41], [46]–[49].

Increased levels of the pyrimidines, uridine and uracil, suggest an inhibition of RNA synthesis (Figure 3(5)). Perhaps the cessation of RNA synthesis offers another ureogenic pathway, albeit much less studied, where uracil is degraded to malonate and urea by bacteria (Figure 3(5a)) [50]. This oxidative pyrimidine degradation pathway has only been identified in microorganisms [50]–[53], which leads us to hypothesize that the nitrogen from uracil may be a good substrate for certain species of the gut microbial community and may aid in internal NH_3_ detoxification. Since metabolomics deals with systems biology and most biological systems are not true single species because of symbiotic relationships, we need to continually consider the response of co-existent species to external environmental stresses [54]–[56]. Furthermore, there is also a reductive pyrimidine pathway that yields NH_3_ and CO_2_ from uracil (Figure 3(5b)). This pathway occurs in a wide variety or organisms from bacteria to humans [53]. If this pathway is active, the NH_3_ produced would be cycled in the OUC to form urea. Literature on this biochemical pathway in crustaceans is non-existent. To conclude whether either pyrimidine degradation pathway is active, confirmation is needed that the active enzymes in these pathways can be identified in shrimp.

The increased levels of amino acids from protein catabolism (Figure 3(1)) suggests possible pathways to alleviate the excess NH_3_, either the deaminases that break down amino acids into NH_3_ are down-regulated and correspond to the increase in FAA for storage [41], [46], [57], or amino acids are being synthesized to reduce the body levels of NH_3_ [57]. The most energetically favorable pathway will likely be the cause of this response.

Mechanisms to survive stressful elevated NH_3_ environments require increased energy. The production of urea through the OUC is an exergonic reaction requiring ATP, which decreased as a result of the nursery TAN spike (Table S3(a)). The potential self-limitation of feed during this stress eliminates much needed nutritionally-derived energy, thus demanding energy from an endogenous source. There was a significant rise in carbohydrate metabolism seen in the form of increased levels of glucose and maltose which can be explained by higher rates of gluconeogenesis [47]. During this critical growth period, metabolism was switched from anabolic to catabolic and into survival mode. The observed high levels of important methyl donors (methionine and choline) signify the discontinuation of macromolecular generation during this stressful event. These findings provide evidence that body mass production is inhibited and actually reversed in this stressful environment.

Growth during the juvenile stage is typically rapid [58]. The metabolites changing with growth are indicative of basic anabolic biochemical processes needed for maturation (Table S3(b)). Proteins and nucleic acids are synthesized with the resultant decrease in amino acids and nucleotides, and energy is produced during nursery growth as indicated by a general increase in carbohydrate levels, ATP/ADP, and lactate. These metabolomic results emphasize the importance of water quality on growth especially during the critical nursery stages of growth. High protein feeds in overabundance are most likely the cause of unfavorable water quality [59].

#### Raceway phase

**Figure 4:**
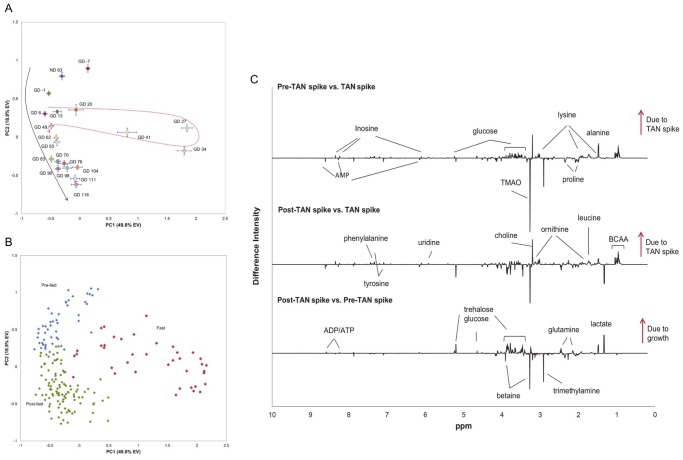
Raceway multivariate statistical analysis. PCA scores plot of muscle tissue from growout phase shrimp from the raceway system. (A) Each point represents the mean PC score for the sampling time point (ND: nursery phase day, GD: growout phase day) and the error bars represent the standard error of the mean. The solid black arrow suggests metabolic changes in relationship to growth, while the dashed red arrow denotes a deviation from and a return to “normal” growth. (B) Scores plot of individual growout phase shrimp colored by pre-fast (blue), fast (red), and post-fast (green). (C) SDS displaying metabolite changes in response to the fasting period and growth. The top spectrum compares pre-fast vs. fast shrimp, the middle spectrum represents fasted shrimp vs. post-fast shrimp, and the bottom spectrum displays metabolite changes from pre-fast vs. post-fast shrimp. Metabolites increasing as a result of the fast are positive in the top two spectra and metabolites increasing during growth are positive in the bottom spectrum. Not all significantly changing metabolites mentioned in the text are annotated here.

The 126 day raceway growout resulted in the collection of 200 individual shrimp that were dissected for muscle tissue. One hundred ninety-three muscle samples were analyzed by NMR and evaluated in the PCA. A trend was evident in the scores plot (PC1 vs. PC2) of the muscle tissue in the PC1 and PC2 dimensions (Figure 4A), most likely representing the normal growth of the shrimp under these conditions. The first two principal components explained almost 70% of the total variance in the data; PC1 and PC2 explained 49.8% and 18.9% of the total variance, respectively. The greatest metabolic changes (PC1) occurred from GD 20 to GD 41. On GD 9, surface scum, presumably resulting from the buildup of waste solids, uneaten food and nutrients, began to accumulate. Feed was withheld starting on GD 14 in order to minimize additional surface scum buildup until the microbial community in conjunction with a settling tank could process and eliminate the waste. The biofloc particulates were the main food source during this period. As the surface scum was cleared from the raceway, pellet feed was gradually added back starting on GD 26 with normal feeding amounts reached by GD 41. The metabolic deviations from “normal” growth coincide with the reduction in feed and/or transition to a diet consisting of biofloc and surface scum residues. Shrimp growth had averaged approximately 1.0 grams per week through GD 20. Between GD 21 and GD 27 the growth rate dropped to −0.35 grams per week and then averaged just 0.49 grams per week between GD 28 and GD 41 (Figure S3B). Both metabolism and growth recovered upon restoring feed and the growth rate averaged 1.38 grams per week for the next eight weeks between GD 42 and GD 97.

Metabolic signatures of the fasting event and raceway growth were determined by SDS plots comparing the shrimp harvested prior to the fasting event (ND 63– GD 13), fasted shrimp (GD 20– GD 41) and the shrimp collected to the end of the growout (GD 48– GD 118; Figure 4B and 4C, Table S3(c–d)). SDS calculations revealed that, compared to pre-fasted shrimp, fasted shrimp (Figure 4C top, FAST minus FAST_pre_ ) had metabolomes generally lower in most amino acids (BCAAs, proline, alanine, serine, asparagine, glutamine, histidine, aspartate, glutamate, phenylalanine, and tyrosine), the organic acid lactate, uridine, and the carbohydrates maltose, glucose, glucose-1-phosphate and trehalose, and had higher levels of glycine, arginine, inosine, carnitine, and ADP/ATP. Similar metabolic changes were identified in the SDS comparing post-fasted shrimp to fasted shrimp (Figure 4C middle, FAST_post_ minus FAST). The metabolite level changes shown in Table S3(c) suggest that energy metabolism was affected and basically the shrimp were in starvation mode. The overall decrease in carbohydrates indicated fuel sources were low and the decrease of nearly all amino acids suggest catabolism of these glucogenic compounds to generate glucose via gluconeogenesis because the exogenous and/or endogenous sources had become insufficient (Table S3(c)). The decreased levels of lactate also indicate contribution to the gluconeogenic supply of glucose for energy needs. Although ADP and ATP are indistinguishable in the shrimp muscle spectra, the increase in ADP would corroborate gluconeogenic activity, while the increase in ATP may be produced from the reduced carbon skeletons of amino acids shuttled through the tricarboxylic acid (TCA) cycle and oxidative phosphorylation pathways. Glycine and arginine are the only amino acids that increased during the fasting. These are the two most abundant amino acids in penaeid muscle tissue [46] and it seems that a sparing mechanism is in place to save the integrity of the muscle during survival mode. Inosine is the only nucleoside that increased significantly during this stress, implying inhibition of DNA synthesis or perhaps an anti-inflammatory response [60].

Muscle protein is the main nutritional reserve in shrimp [61] and this is evident in the above analyses, but lipid metabolism has been suggested as the first energy source during starvation in shrimp [62], [63]. In this metabolomic study, we focused our attention on the polar metabolites. Although we did not specifically assess the lipid metabolome, we were still capable of identifying biochemical compounds involved in this energy sequestration pathway. Carnitine, which is increased in the fasted muscle tissue, is a marker of fatty acid metabolism. Its main function is to transport fat into the mitochondria of muscle cells for oxidation, ultimately producing energy from the tricarboxylic acid cycle [64].

Studies have shown biofloc can be a supplement to traditional protein-based feeds[59], [65]–[70] but bioflocs do not allow for complete nutritional replacement [71]. This metabolomics analysis clearly shows that relying on biofloc as the sole shrimp feed cannot sustain a healthy, growing population. To survive this unfavorable feeding condition, energy is derived from protein turnover, gluconeogenesis, and lipid metabolism to maintain vital processes.

In an intensive growing environment, any stressor affecting the physiological health of an organism gives rise to the concern of disease susceptibility and compromised immune condition. No outbreak of disease or illness following the fasting event was noted. It has been shown that shrimp fed an optimal diet rich in protein prior to the deprivation period maintained immunity for a week longer than shrimp fed a restricted protein diet and were able to physiologically tolerate the starvation for a longer period of time [62]. Biofloc may also have contributed to protection from disease or pathogens during this stress [72], [73].

Once feed was reinstated at normal levels, shrimp were able to recover from the deprivation period, both metabolically and in growth. Zhang and colleagues (2010) found that as the period of starvation increased, the reestablishment of normal food intake levels during recovery feeding were delayed, but feed conversion efficiency was higher resulting in a rapid growth spurt that restored levels equivalent to the controls[74]–[76]. This compensatory growth is an adaptation to tolerate moderate periods of food deprivation.

Physiological changes due to growth were observed in comparing pre-fasted shrimp versus post-fasted shrimp identifying increased levels of lactate, arginine, dimethylamine, TMAO and proline, and decreased levels of amino acids, acetoacetate, choline, sarcosine, uridine, AMP, ADP/ATP and betaine in the shrimp ready for harvest (Figure 4C bottom, Table S3(d), FAST_post_ minus FAST_pre_). As shrimp mature, growth becomes asymptotic [58] where anabolic metabolism has slowed and maintaining homeostasis has become the focus. Protein turnover and energy production have reached a steady state.

In comparing the levels of metabolic change occurring in the raceway growth with changes in the nursery growth (Table S3) and the effect of stressors during each production phase, stress during the early stages of growth had a greater metabolic response. This is most likely due to the fact that anabolic development should have been the priority at this life stage and metabolism had to completely shift to detoxification and survival to cope with the ammonia stress. Tolerance to ammonia increases as larvae develop to juveniles in penaeid shrimp [39], [77] and consequently explains the absence of metabolic stress observed in the muscle tissues during a raceway TAN spike on GD27-GD34 and GD83. We must also consider that the nursery analysis consisted of whole body organisms and thus we observed a tissue-averaged metabolism, whereas, the muscle tissue provided only a compartmental (mainly energetic) view of the overall metabolism. Other events, like the NO_2_-N spikes in the raceway growout, did not have metabolic effects on the muscle tissue analyzed here, however such stressors may have a greater effect (than TAN and starvation) in other tissues and could provide a more complete understanding of the physiological responses of shrimp to different conditional changes experienced throughout the growout.

#### Stocking stress

**Figure 5:**
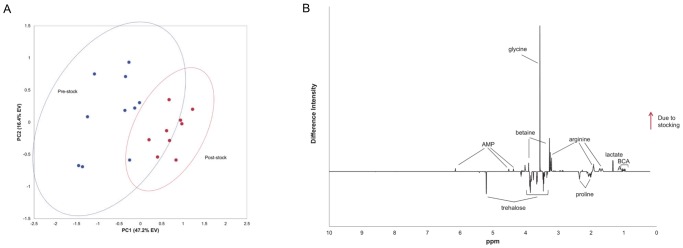
Stress of stocking. (A) Raceway stocking stress PCA scores plot of muscle tissue. Sampling from one day pre-stock (ND 63) and one day Post-stock (GD -7). Ellipses represent 95% confidence intervals. (B) SDS of metabolites changing in response to the stress of stocking from the nursery to the raceway. Metabolites increasing as a result of the stocking are positive and metabolites decreasing are negative.

In the raceway growout analysis above, sampling time point ND 63 from the nursery phase was included because at that time the shrimp had matured from PL to juveniles and were too large to collect as whole-body biological composites, but were easily dissected for comparable tissues. These samples were taken a day prior to the primary raceway stocking and GD -7 shrimp were sampled the day following stocking, allowing us to analyze for stress from the stocking event (Figure 5A). The SDS from the raceway stocking PCA model (pre- vs. post-stocking) exhibited lower levels of proline, maltose, glucose and trehalose, UDP-glucose and higher levels of glycine, N,N-dimethylglycine, lactate, BCAAs, asparagine, arginine, choline, betaine, carnitine, glucose-1-phosphate and AMP in response to the stocking (Figure 5B, Table S3(e)). These results are typical and well known as handling stress responses for crustaceans [78]–[80]. The decrease in muscle carbohydrates and increase in lactate level indicate glycolytic fuel production. Energy is also produced by glycogenolysis as seen in the increase of glucose-1-phosphate produced from glycogen degradation. Increased carnitine suggests the use of lipids as an energy source. Phospholipid metabolism was apparent with the increase of the choline oxidation pathway which increased levels of choline, betaine, N,N-dimethylglycine and glycine. The increase of the essential amino acids arginine, isoleucine, leucine and valine correspond to protein degradation. Interestingly, inosine was increased in response to this stocking stress, as well as in response to the stressors in the nursery and raceway growout, where trehalose was decreased in all three stressful events. Inosine and trehalose may be useful biomarkers of general stress in *L. vannamei*.

Although efforts were made to minimize the handling stress of transferring juveniles from the nurseries to the growout raceway, this gentle stocking still induced an increased metabolic demand on the shrimp. Perhaps the trauma of transport, and/or being introduced into a new environment, and/or stress-induced food deprivation may have caused the metabolic disturbance. In order to achieve optimally successful production, minimizing all stressors to the greatest extent possible is imperative.

#### Summary

In this metabolomic health assessment of shrimp through a complete growout, a wealth of biochemical information was gained from common physiologically stressful growing conditions. Although additional studies are needed to validate these results, we can propose recommendations in management practices based on this data to convey the utility of metabolomics for optimal aquaculture production. For example, a farmer could routinely monitor stress levels with inosine and/or trehalose test kits to ensure his crop is growing with minimal levels of stress and also provide an early detection method to a potentially detrimental problem so that remediation can occur before the situation reaches a state that may affect water quality, feed consumption or growth. Metabolomics can be used to define what levels of various parameters (dissolved oxygen (DO), pH, NH_3_, NO_2_, NO_3_, etc.) induce a metabolic stress reaction that is not otherwise detectable, and thereby greatly improve protocols for cultivation. Perhaps special diets formulated with higher carbohydrate and lipids, fed prior to stocking, can mitigate the stress of transfer by building up glycogen stores beforehand. Similarly, a higher carbohydrate diet may provide the nutritional energy required to endure a water quality stress like rising levels of TAN.

### Conclusion

Aquaculture involves a delicate balance of feed, water quality and organism physiology to achieve commercially viable crops. This study biochemically assessed the health of superintensively produced shrimp with NMR-based metabolomics. We identified tissue specific biochemical changes in response to feeding and water quality events during this growout. The metabolic changes identified largely pertain to energy metabolism and nitrogen detoxification. These are crucial biochemical pathways that effect growth. Management to maintain physiological homeostasis is essential in an industry that desires rapid, nutrient rich crop production. This research highlights one aspect of the usefulness of NMR-based metabolomics in aquaculture.

## Supporting Information

Figure S1 **Data quality assurance.** Quality assessment of the extraction protocol. (A) PCA scores plot of all extracted shrimp muscle (•), shrimp muscle control material (CM, •), and NIST standard reference material (SRM, •) showing the extraction variability is minimal compared to the individual variability of the shrimp. (B) PCA scores plot of the triplicate samples and the CM and SRM samples showing the triplicate extraction and analysis variability in PC space. (C) ^1^H NMR spectra of six extraction blanks (top six) and the NMR buffer (bottom). The arrow indicates a contaminant from one brand of bead beating tubes. This peak was excluded from statistical analyses.
(TIF)

Figure S2 **Annotated ^1^H NMR shrimp spectra.** Representative ^1^H NMR proton spectra of shrimp polar metabolites from the nursery composites (n = 1 to 5) (top) and raceway individual muscle samples (bottom) from the growout aquaculture systems. (A) upfield NMR spectrum (0 to 4 ppm), (B) downfield NMR spectrum (4 to 10 ppm). (1) leucine, (2) valine, (3) isoleucine, (4) lactate, (5) alanine, (6) lysine, (7) arginine, (8) proline, (9) glutamine, (10) methionine, (11) glutamate, (12) acetoacetate, (13) aspartate, (14) dimethylamine, (15) sarcosine, (16) asparagine, (17) ornithine, (18) O-acetylcarnitine, (19) choline, (20) TMAO, (21) glucose, (22) trehalose, (23) glycine, (24) threonine, (25) betaine, (26) serine, (27) inosine, (28) AMP, (29) ADP, (30) maltose, (31) uracil, (32) uridine, (33) UDP-glucose, (34) adenosine, (35) ATP, (36) fumurate, (37) 3-hydroxykynurenine, (38) kynurenine, (39) tyrosine, (40) histidine, (41) tryptophan, (42) phenylalanine, (43) Τ-methylhistidine, (44) guanosine, (45) adenine, (46) formate. This is not a complete list of annotated compounds from shrimp spectra, see Table S2.
(TIF)

Figure S3 **Shrimp growth.** Growth curves from (A) the Nursery phase and (B) the Growout phase presented as means with ±1 standard error of the mean.
(TIF)

Figure S4 **Visual for nursery metabolite changes.** Nursery shrimp metabolite responses to a TAN spike event and time shown as relative integrated peak area for (A) amino acids and (B) nucleosides and nucleobases.
(TIF)

Table S1 **Water quality parameter summary.** The mean ± one standard deviation is provided for each parameter.
(DOC)

Table S2 **Identified shrimp metabolites.** Compound list of annotated metabolites identified in shrimp from the nursery through the growout phase.
(DOC)

Table S3 **Significant shrimp metabolite changes in response to events that occurred during the aquaculture growout (Nursery TAN spike, Raceway fasting event, and Raceway stocking) and also with growth over time during both Nursery and Raceway growouts.**
(DOC)
