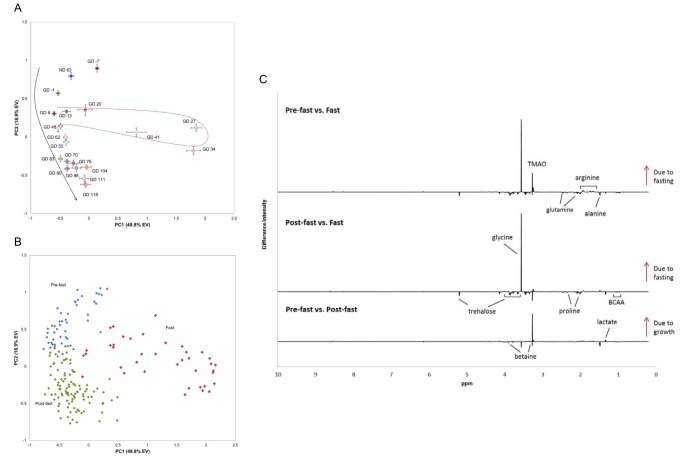# Correction: Evaluation of Pacific White Shrimp (*Litopenaeus vannamei*) Health during a Superintensive Aquaculture Growout Using NMR-Based Metabolomics

**DOI:** 10.1371/annotation/71a874d8-881f-4716-83ce-7af9fffb5462

**Published:** 2013-11-12

**Authors:** Tracey B. Schock, Jessica Duke, Abby Goodson, Daryl Weldon, Jeff Brunson, John W. Leffler, Daniel W. Bearden

Figure 4C was erroneously published as a duplicate of Figure 2C. Please see the corrected Figure 4 here: 

**Figure:**