# Supplementary figures and images for: Evaluation of Pacific White Shrimp (Litopenaeus vannamei) Health during a Superintensive Aquaculture Growout Using NMR-Based Metabolomics
Source: PLoS One. 2013 Mar 26;8(3):e59521. doi: 10.1371/journal.pone.0059521 (PMC3608720; doi:10.1371/journal.pone.0059521)

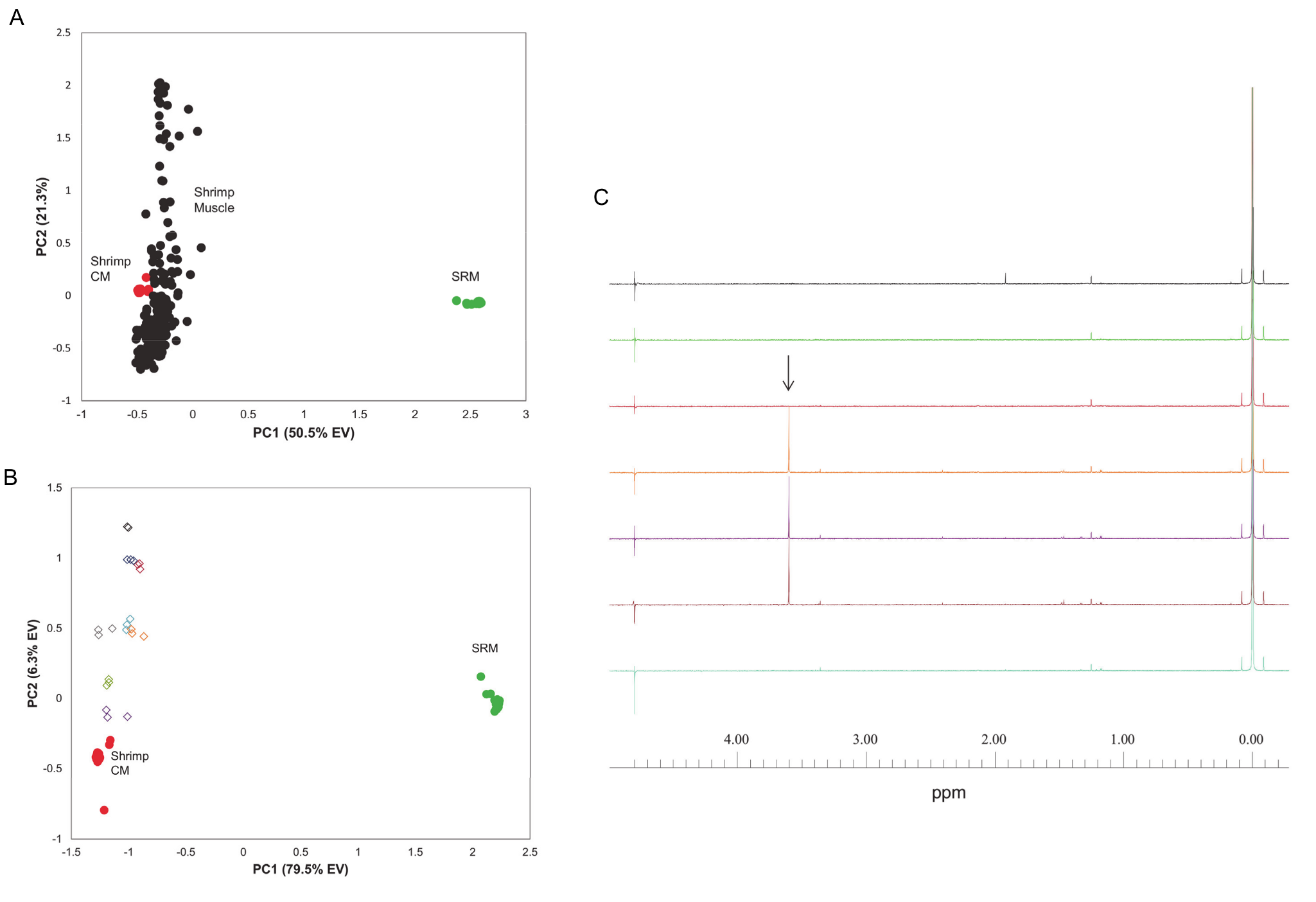

Supplement: Figure S1 — Data quality assurance. Quality assessment of the extraction protocol. (A) PCA scores plot of all extracted shrimp muscle (•), shrimp muscle control material (CM, •), and NIST standard reference material (SRM, •) showing the extraction variability is minimal compared to the individual variability of the shrimp. (B) PCA scores plot of the triplicate samples and the CM and SRM samples showing the triplicate extraction and analysis variability in PC space. (C) 1H NMR spectra of six extraction blanks (top six) and the NMR buffer (bottom). The arrow indicates a contaminant from one brand of bead beating tubes. This peak was excluded from statistical analyses. (TIF) [file pone.0059521.s001.tif]

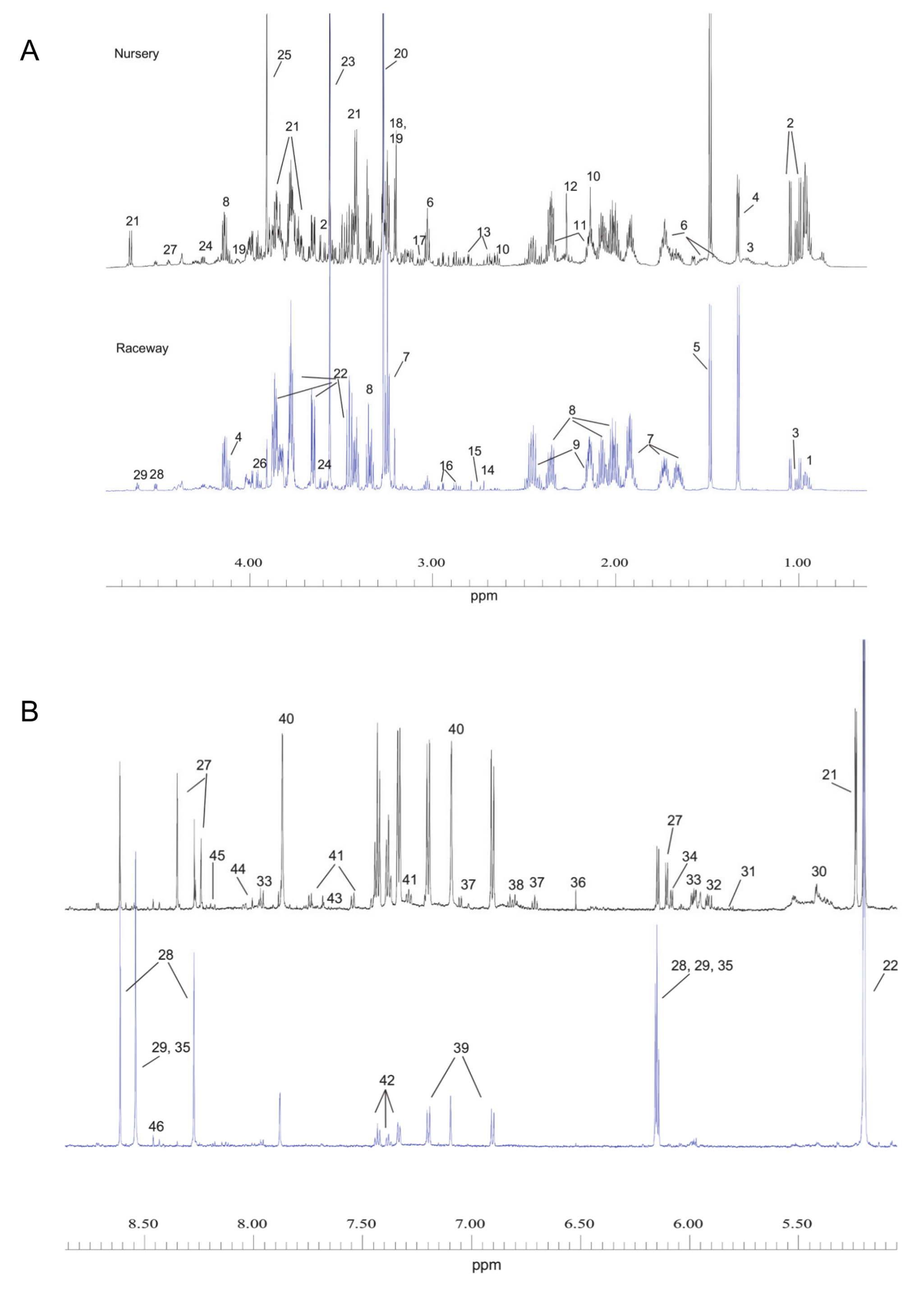

Supplement: Figure S2 — Annotated 1H NMR shrimp spectra. Representative 1H NMR proton spectra of shrimp polar metabolites from the nursery composites (n = 1 to 5) (top) and raceway individual muscle samples (bottom) from the growout aquaculture systems. (A) upfield NMR spectrum (0 to 4 ppm), (B) downfield NMR spectrum (4 to 10 ppm). (1) leucine, (2) valine, (3) isoleucine, (4) lactate, (5) alanine, (6) lysine, (7) arginine, (8) proline, (9) glutamine, (10) methionine, (11) glutamate, (12) acetoacetate, (13) aspartate, (14) dimethylamine, (15) sarcosine, (16) asparagine, (17) ornithine, (18) O-acetylcarnitine, (19) choline, (20) TMAO, (21) glucose, (22) trehalose, (23) glycine, (24) threonine, (25) betaine, (26) serine, (27) inosine, (28) AMP, (29) ADP, (30) maltose, (31) uracil, (32) uridine, (33) UDP-glucose, (34) adenosine, (35) ATP, (36) fumurate, (37) 3-hydroxykynurenine, (38) kynurenine, (39) tyrosine, (40) histidine, (41) tryptophan, (42) phenylalanine, (43) Τ-methylhistidine, (44) guanosine, (45) adenine, (46) formate. This is not a complete list of annotated compounds from shrimp spectra, see Table S2. (TIF) [file pone.0059521.s002.tif]

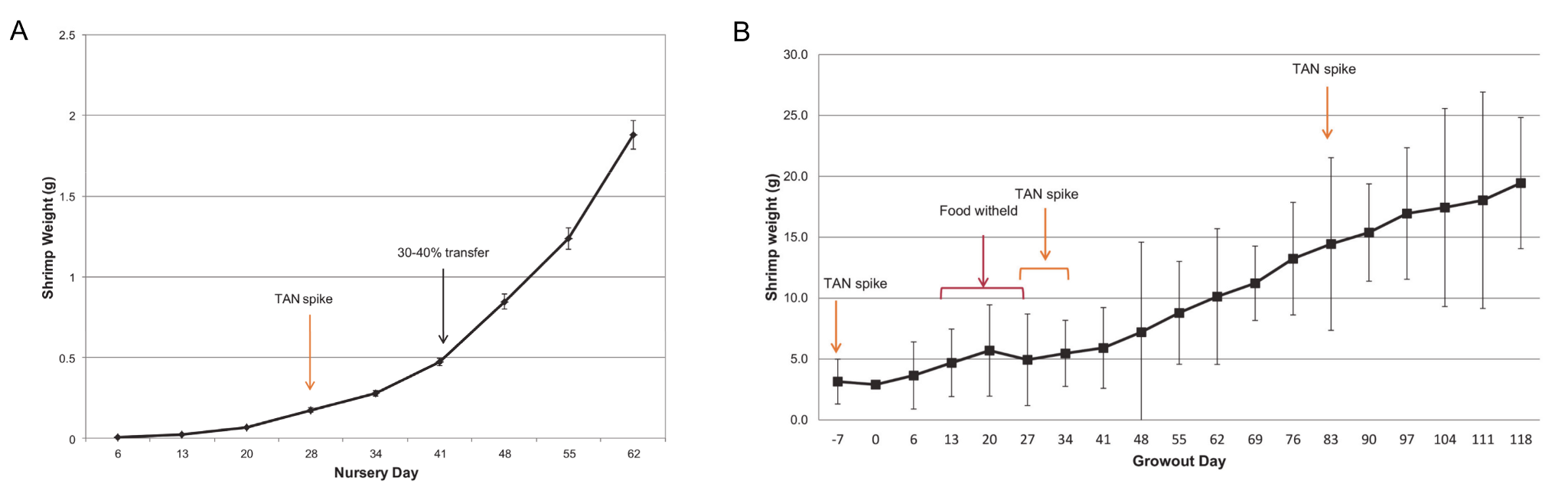

Supplement: Figure S3 — Shrimp growth. Growth curves from (A) the Nursery phase and (B) the Growout phase presented as means with ±1 standard error of the mean. (TIF) [file pone.0059521.s003.tif]

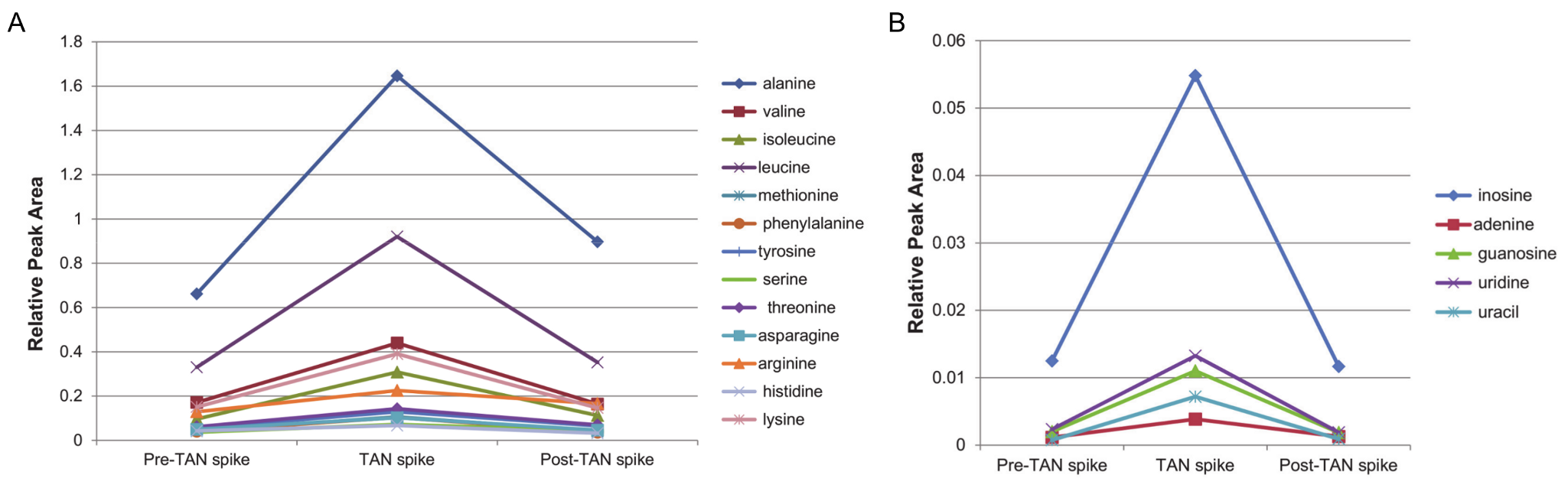

Supplement: Figure S4 — Visual for nursery metabolite changes. Nursery shrimp metabolite responses to a TAN spike event and time shown as relative integrated peak area for (A) amino acids and (B) nucleosides and nucleobases. (TIF) [file pone.0059521.s004.tif]
