# Supplementary material for: Evaluation of Pacific White Shrimp (Litopenaeus vannamei) Health during a Superintensive Aquaculture Growout Using NMR-Based Metabolomics
Source: PLoS One. 2013 Mar 26;8(3):e59521. doi: 10.1371/journal.pone.0059521 (PMC3608720; doi:10.1371/journal.pone.0059521)
Supplement: Table S1 — Water quality parameter summary. The mean ± one standard deviation is provided for each parameter. (DOC) [file pone.0059521.s005.doc]

**Table S1. Water quality parameter summary.**

**
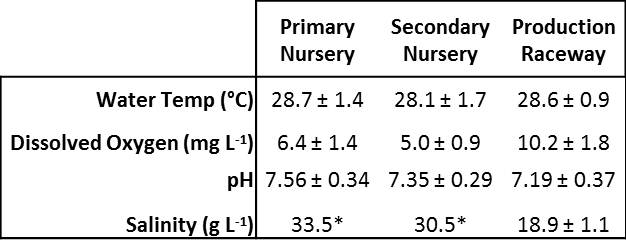
**

* denotes salinity measured at initial stocking

The mean ± one standard deviation is provided for each parameter.
