# Supplementary material for: Evaluation of Pacific White Shrimp (Litopenaeus vannamei) Health during a Superintensive Aquaculture Growout Using NMR-Based Metabolomics
Source: PLoS One. 2013 Mar 26;8(3):e59521. doi: 10.1371/journal.pone.0059521 (PMC3608720; doi:10.1371/journal.pone.0059521)
Supplement: Table S2 — Identified shrimp metabolites. Compound list of annotated metabolites identified in shrimp from the nursery through the growout phase. (DOC) [file pone.0059521.s006.doc]

**Table S2. Identified shrimp metabolites.**

| 2-Aminoadipate | | Malate | |
| --- | --- | --- | --- |
| 2'-Deoxyadenosine | | Malonate | |
| 2'-Deoxyinosine | | Maltose | |
| 2-Hydroxyglutarate | | Mannose | |
| 3-Hydroxybutyrate | | Methionine | |
| 3-Hydroxykynurenine | | Methylamine | |
| Acetoacetate |  | | N,N-Dimethylglycine |
| Adenine |  | | NAD+ |
| Adenosine |  | | Nicotinate |
| ADP |  | | O-Phosphocholine |
| Alanine |  | | Ornithine |
| AMP |  | | Phenylalanine |
| Arginine |  | | Proline |
| Asparagine |  | | Pyroglutamate |
| Aspartate |  | | Sarcosine |
| ATP |  | | Serine |
| Betaine |  | | Succinate |
| Carnitine |  | | Taurine |
| Choline |  | | Threonate |
| Creatine |  | | Threonine |
| Creatinine |  | | T-Methylhistidine |
| Dimethylamine | | Trans-4-Hydroxy-L-proline | |
| Formate |  | | Trehalose |
| Fumarate |  | | Trimethylamine |
| Glucose |  | | Trimethylamine N-oxide |
| Glucose-1-phosphate | | Tryptophan | |
| Glutamate |  | | Tyrosine |
| Glutamine |  | | UDP-galactose |
| Glycine |  | | UDP-glucose |
| GTP |  | | Uracil |
| Guanosine |  | | Uridine |
| Histamine |  | | Valine |
| Histidine |  | | Xanthine |
| Homarine |  | |  |
| Inosine |  | |  |
| Isoleucine |  | |  |
| Kynurenine |  | |  |
| Lactate |  | |  |
| Leucine |  | |  |
|  |  | |  |

Compound list of annotated metabolites identified in shrimp from the nursery through the growout phase.
