# Supplementary material for: Evaluation of Pacific White Shrimp (Litopenaeus vannamei) Health during a Superintensive Aquaculture Growout Using NMR-Based Metabolomics
Source: PLoS One. 2013 Mar 26;8(3):e59521. doi: 10.1371/journal.pone.0059521 (PMC3608720; doi:10.1371/journal.pone.0059521)
Supplement: Table S3 — Significant shrimp metabolite changes in response to events that occurred during the aquaculture growout (Nursery TAN spike, Raceway fasting event, and Raceway stocking) and also with growth over time during both Nursery and Raceway growouts. (DOC) [file pone.0059521.s007.doc]

**Table S3.** **Significant shrimp metabolite changes in response to events that occurred during the aquaculture growout (Nursery TAN spike, Raceway fasting event, and Raceway stocking) and also with growth over time during both Nursery and Raceway growouts.**

**
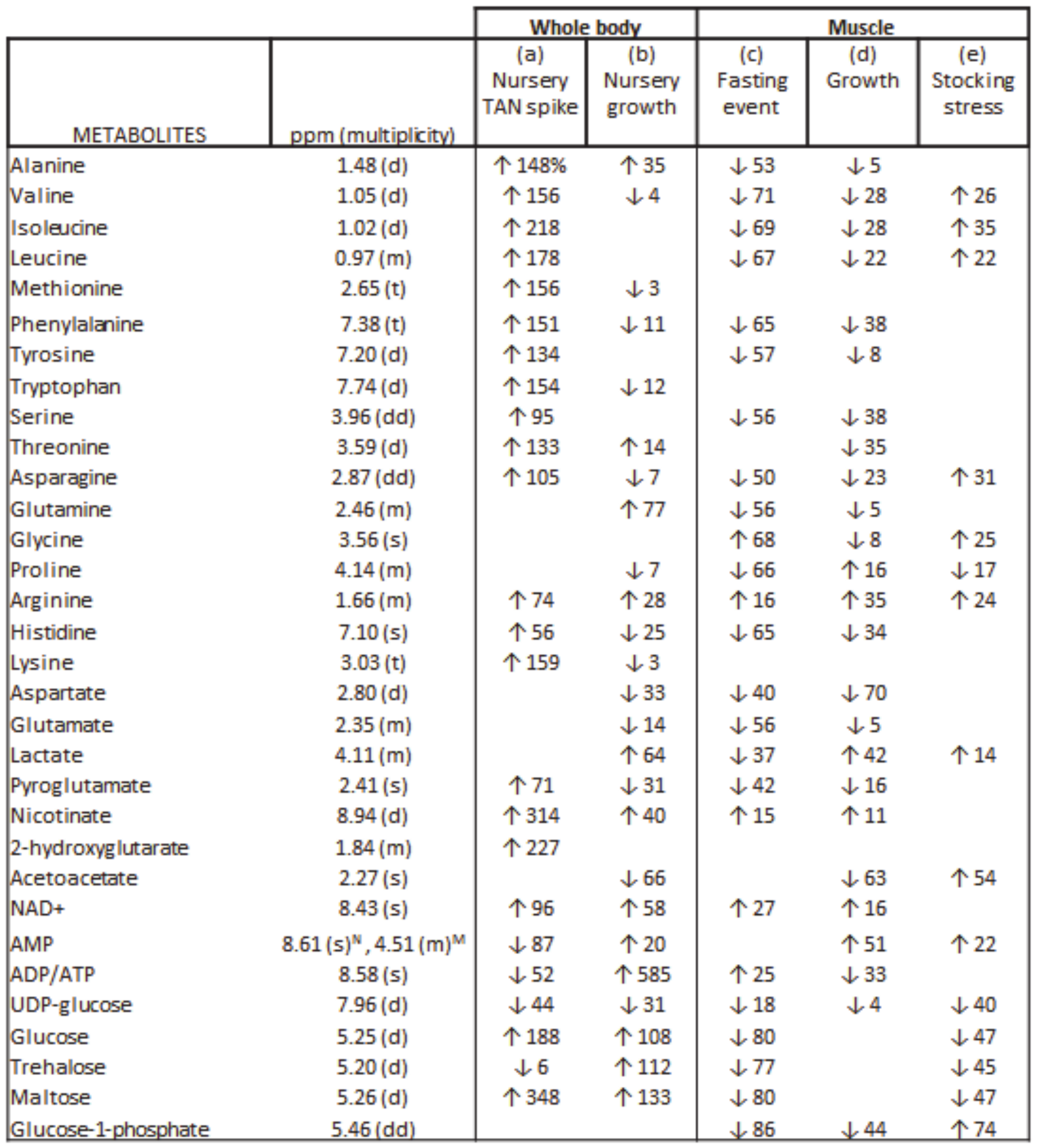
**

**
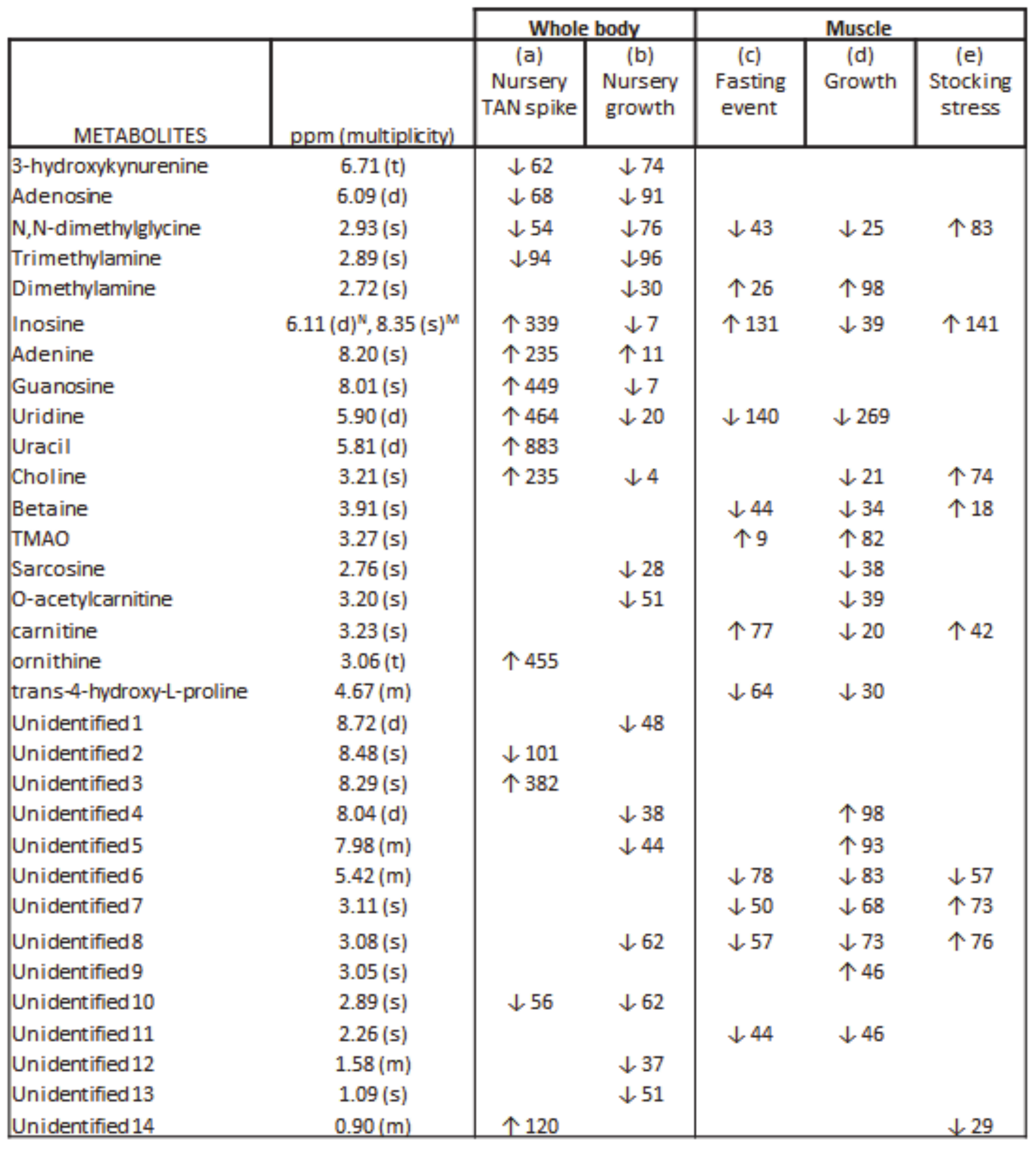
**

Arrows indicate direction of change relative to the event or with time. The relative percent change of the integrated peak area is listed next to the arrows.

^N^ NMR chemical shift used for metabolite integration for the Nursery shrimp composite spectra.

^M^ NMR chemical shift used for metabolite integration for the Raceway shrimp muscle pectra.

Otherwise, the chemical shift noted was similar for both shrimp growout stages.
